# P-247. Visceral Leishmaniasis Subcohort HIV General Cohort Descriptive Observational Study 2010–2024

**DOI:** 10.1093/ofid/ofaf695.469

**Published:** 2026-01-11

**Authors:** Pedro A Villalba apestegui, Gustavo-Adolfo Méndez, Carla Niveyro, lucila maria COMPAÑY KEC, Cynthia Tomasino, Ricardo de Jesus Solari Maidana

**Affiliations:** Infectious Diseases Department, Hospital Escuela de Agudos Dr. Ramón Madariaga. Posadas, Misiones – Argentina, posadas, Misiones, Argentina; Hospital Escuela de Agudos Dr Ramón Madariaga, Posadas, Misiones, Argentina, Posadas, Misiones, Argentina; Madariaga Hospital, Posadas, Misiones, Argentina; Infectious Diseases Department, Hospital Escuela de Agudos Dr. Ramón Madariaga. Posadas, Misiones – Argentina, posadas, Misiones, Argentina; Infectious Diseases Department, Hospital Escuela de Agudos Dr. Ramón Madariaga. Posadas, Misiones – Argentina, posadas, Misiones, Argentina; Infectious Diseases Department, Hospital Escuela de Agudos Dr. Ramón Madariaga. Posadas, Misiones – Argentina, posadas, Misiones, Argentina

## Abstract

**Background:**

Visceral leishmaniasis in people living with HIV (PLHIV) is a life-threatening opportunistic infection in endemic regions, yet South American cohorts remain poorly characterized. Despite improvements in ART access, visceral leishmaniasis remains a neglected opportunistic infection in PLHIV in endemic areas of South america, with limited longitudinal cohorts assessing PCR-based diagnosis and immunovirologic predictors of outcome.

**Methods:**

We conducted a retrospective descriptive cohort study of adult PLHIV with laboratory-confirmed VL (bone marrow microscopy and/or PCR) between Jan 2010–Dec 2024. Data collected included age, sex, ART status, CD4⁺ T-cell count, CD4%, HIV viral load, hematocrit, platelet count, serum glucose, urea and creatinine, and bone marrow PCR results. Descriptive statistics (medians, interquartile ranges, proportions) summarized the cohort; we compared key variables between patients who died and those who survived.

**Results:**

Fourteen male patients (median age 42 years [IQR 22–63]) met inclusion criteria. At diagnosis, median CD4 count was 86.92 cells/µL (IQR 5–278), CD4% 15.9% (IQR 3.0–58), and HIV viral load 800.023 copies/mL (IQR 75–3,020,000); none had undetectable viremia. Seven of 14 (50%) were on ART. Median hematocrit was 25.4% (IQR 16–37) and platelet count 80,808/µL (IQR 18,320–220,000). Bone marrow PCR was positive in 75% of those who died and 71% of survivors. Overall mortality was 28.6% (4/14). Compared with survivors, patients who died had higher median viral loads (3,020,000 vs 522,526 copies/mL), lower ART coverage (25% vs 60%) and more severe thrombocytopenia (median 52,000 vs 92,332/µL).

**Conclusion:**

PLHIV with visceral leishmaniasis at our center exhibit advanced immunosuppression, significant hematologic derangements and suboptimal ART coverage. High HIV viral load, ART-naïve status and severe cytopenias emerged as potential predictors of mortality, reinforcing the need for prompt ART initiation and close hematologic monitoring.Early ART initiation and incorporation of PCR into routine VL work-up could reduce mortality in PLHIV. Prospective studies are needed to validate these predictors and optimize integrated HIV–leishmaniasis care pathways

**Disclosures:**

All Authors: No reported disclosures

